# “I like the way you move”: how hormonal changes across the menstrual cycle affect female perceptions of gait

**DOI:** 10.1186/1756-0500-5-453

**Published:** 2012-08-21

**Authors:** Rick van der Zwan, Natasha Herbert

**Affiliations:** 1 Psychology, Southern Cross University, Coffs Harbour, NSW, Australia

## Abstract

**Background:**

Variations in hormone concentrations across the menstrual cycle affect human female mate preferences. It has been shown that around the time of ovulation human females prefer more masculine male voices, faces, and bodies while simultaneously preferring less faces that are more feminine. They prefer also displays of male dominance, males with more symmetrical faces, and the scent of males with high levels of body symmetry. The aim of the experiments reported here was to investigate whether there are changes in female preferences for walking gaits across the menstrual cycle.

**Results:**

Experiment 1 showed female observers could discriminate between point-light walkers with low and high levels of fluctuating asymmetries in their gaits. Female observers were more sensitive to asymmetries in female gaits than they were for asymmetries in male gaits. Experiment 2 showed that level of gait asymmetry did not affect the abilities of observers to discriminate female from male walkers. Experiment 3 showed that female observers did not change their preference for low and high asymmetry walkers across their menstrual cycles. However, females showed a decreased preference for all female walkers at the time during which it was estimated observers were at peak fertility. That same change in preference was not observed for male walkers.

**Conclusions:**

These data suggest female observers may not value gait asymmetry, as a mate selection cue, in the same way that they value asymmetries in faces and bodies. While only “average” gaits were used in these experiments, rather than the gaits of individual walkers, the types of asymmetries in gait tested here were not used in the same way as static cues for judging the apparent healthiness of individuals. Females do discriminate well average female gait asymmetries and do change their preferences for those gaits across their menstrual cycle. Doing so may reflect the operation of processes that equip females with an advantage when competing for mates at times of peak fertility.

## Background

Variations in hormone concentrations across the menstrual cycle affect human female mate preferences. It has been shown that during the stage of the menstrual cycle during which women are most likely to become pregnant from a single act of sexual intercourse (the high conception risk, HCR, phase) that they prefer more masculine male voices [1], faces [2-6], and bodies [7,8]. During HCR females also show preferences for increased dominant male behavioural displays such as direct intrasexual competitiveness [9], for male faces with low levels of facial asymmetry [10], and for the scent of men with low levels of body asymmetry [11]. Simultaneously, females show decreased preferences for more feminine faces during HCR [3]. That is, HCR signals preference changes for both male and female stimuli.

The relationship between HCR and preferences for more, rather than for less symmetry are interesting. Every human body and face has some level of fluctuating asymmetry (FA), manifesting as deviations from perfect bilateral symmetry. Usually FA is low with individuals accumulating only small asymmetries during development. Indeed, resistance to environmentally induced FA is thought to indicate underlying genetic strength [12]. Perhaps because of that, preferences for low levels of FA have been observed for bodily [13] and facial features [14-17] in human and even non-human observers [18]. Interestingly, human individuals with low FA are reported to have better emotional and psychological health [19], have lower rates of mortality and morbidity [13], and increased fertility [20].

Individuals with low levels of FA are also going to be more symmetrical in their movements. Those with structural asymmetries will typically move asymmetrically: One leg substantially shorter than the other will lead, for example, to a limp. As such, biological motion (BM) has also become a social cue usefully exploited by both female and male observers. Humans are, for example, very good at discriminating the sex of others [21,22], emotional state [23], and even intent [24] from very short presentations of the BMs of others.

With that in mind, it is unsurprising that there is evidence that females use BM as a cue for judging mate potential [8,13]. To date, however, there has been no systematic investigation of the effect of hormonal variations on preferences for, or sensitivities to asymmetries in gait. The present study aimed directly to investigate whether there are changes in female preferences for walking gaits across the menstrual cycle. In particular, the experiments reported here tested the hypothesis that more symmetrical male gaits are preferred by female observers at HCR. Simultaneously, it was predicted also that if there were changes in preferences for female gaits, those too would be observed at HCR.

## Methods

### Statement of ethical approval

The experiments described here were conducted using methods approved by the formally constituted Human Research Ethics Committee (HREC) at Southern Cross University (approval ECN.10.130). The HREC approval ensures these experiments were conducted in accordance in compliance with the Helsinki Declaration.

### Participants

Experiments 1 and 2: Twenty-six premenopausal females (mean age = 22.23, S.D. = 5.44) participated in these two experiments. Participants were students at the University of Southern Cross. All identified themselves as heterosexual and between the ages of 18-36. All had normal or corrected-to-normal vision.

Experiment 3: 16 premenopausal females met inclusion criteria on initial testing: They reported to be between the ages of 18-36 years old; had regular monthly menstrual cycles (always between 28-31 days); were not currently using any form of hormonal contraception or had not been using any hormonal contraception in the last three months; and identified as heterosexual. Of the original 16, four were removed due to irregular menstrual cycles (e.g. menstruating twice in a month) during the course of the experiment. Thus, 12 naturally ovulating women were included in the repeated measures analyses (mean age = 25.25, S.D. = 6.89).

### Stimuli

**Figure 1:**
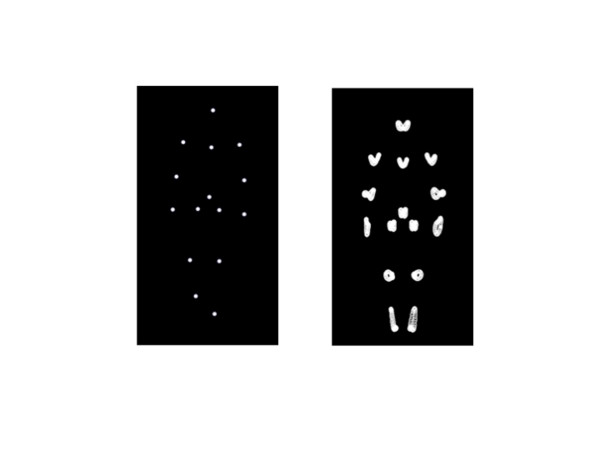
**Examples of the point-light walkers used in these experiments.** Left Panel: An example of the 15 element point-light display used for the experiments reported here. This is the +1 figure from the continuum. The elements delineate the major joints of the human body (shoulders, elbows, wrists, knees, ankles, hips) plus the head, sternum, and mid-abdomen. Right Panel: Depiction of the motion path for each element represented in the Left Panel. Note the asymmetries in the motion paths of elements on each side of the body (most obvious in the ankles, elbows, and wrists). Those asymmetries are ‘average’ asymmetries derived from 100 walkers used in the construction of the models. Note too that the asymmetries are not visible until the model moves.

Custom designed software (PointLightLab, version 4.0.13) was used to create point-light walkers, each composed of 15 point lights. Each point-light represented a major joint on the human body plus the centre of the sternum and hips, Figure 1, Left Panel. It is important to note here that structural information is not eliminated in the type of point-light display used here, but it is reduced. Cues to characteristics like sex and attractiveness (shoulder-to-hip ratio for example) still can be discerned [21] although they are much less salient than in other types of representation.

With that in mind, the stimuli used here were based on “averaged” walkers developed by Troje [25,26]. Troje recorded the natural gaits of 100 individuals (50 female, 50 male) and then computed an ‘average’ human gait. From that average Troje constructed a “gender” continuum of 13 walkers. The 13 increment continuum depicts a range of gaits extending from extreme feminine (-6 on the continuum) to extreme masculine walkers (+6 on the continuum) [26]. Of those 13 increments, 9 point-light walkers were used here: Position -4 represented the most exaggerated female walker used. Position +4 represented the most exaggerated male walker. Position 0 remained the mathematically average or ‘gender ambiguous’ walker.

Each point-light walker from the continuum contained an average amount of fluctuating asymmetry (Figure 1, Right Panel). Such walkers, without alteration, represented the low FA walkers in the experiments reported here (that is, walkers with low asymmetries in gait). To create the high FA walkers (walkers with high asymmetries in gait) new models were created based on the original 9 walkers (between ±4): Each walker has 6 pairs of point-lights marking the shoulders, elbows, wrists, hips, knees and ankles. To create walkers with high FA, one element from each pair was randomly replaced by an element from a walker ±2 increments away along the continuum. For example, the -1 walker (the first walker on the continuum with statistically ‘female’ characteristics) would maintain its normal midline dot points. For each of the remaining 6 pairs however, one dot point would remain as per the -1 walker, and one would contain a dot point from either the -3 walker or the +1 walker.

### Procedure

Participants were invited to take part in the experiment via email invitations and announcements in lectures. Upon arrival at the lab, participants were given information about the experiment in which they were taking part and then each signed an “informed consent” form indicating they knew what was required of them, the use to which the data gathered would be put, and of any risks involved. Participants filled out a preliminary questionnaire asking about their age, sexual orientation, and relationship status. They also provided details of their menstrual cycle, their typical cycle length, and any hormonal contraceptive use. Although menstrual cycle and hormonal contraception information was not used in the data analysis for Experiments 1 and 2, it was gathered to facilitate follow-up, for Experiment 3, with participants who were not on hormonal contraceptives.

The experiment was conducted in a light and sound attenuated room. Stimuli was presented using a Pentium 4 processor paired with a Dell Triniton Flat Screen monitor, display resolution of 1024 × 768 pixels, set at a refresh rate of 100 Hz with 32 bit colour resolution. Participants indicated their responses via key-presses on a Compaq KB-0133 computer keyboard. Viewing distance of the monitor and keyboard was set to 57 cm so that 1 cm on the screen was equivalent to 10˚ of visual angle for observers.

Experiments 1 and 2: Participants were required to do the experiment on two separate occasions, 2 weeks apart. On each occasion they completed 2 blocks of trials, separated by a 5 minute break. Each block consisted of 90 trials. Each trial presented to participants, in random order, a single walker from the continuum of 9 walkers. Four walkers were female (-4, -3, -2, -1), four were male (1, 2, 3, 4), and one was sexually neutral (the mathematic average of all female and male walkers; 0). Each walker was presented with one level (low or high) of fluctuating asymmetry. Thus, in each block 45 trials presented low FA walkers (5 of each increment along the continuum) and 45 trials presented high FA walkers. Each exposure lasted 3 seconds during which the walker completed approximately 4 steps. The total duration of the experiment on any one occasion was less than 15 minutes. In experiment 1, participants were instructed to indicate, on every trial, whether they thought the gait of the walker onscreen was “symmetrical” or “not symmetrical”. The definition of symmetrical provided was, “Does the motion of one side of the body match or ‘mirror’ the motion of the other side?”. In experiment 2, and using the same paradigm, participants indicated whether they thought the walker was “female” or “male”.

Experiment 3: Following previous studies [4-6], a standardised 28-day menstrual cycle model was used to allocate females into two menstrual cycle phases for testing. Ovulation occurs around day 14 in a 28-day menstrual cycle (day 1 is the first day of menstruation). Women experience a peak in oestrogen concentration at this time and it is considered to be the period in a woman’s menstrual cycle that she is most likely to become pregnant with one act of sexual intercourse [27]. Thus, estimated HCR was defined here as days 12-16. In contrast, estimated LCR was considered to be days 26-28, and days 1-3. Conception risk for each participant was calculated using a forward counting method based on reported day of onset of menstruation. That is, HCR was estimated to be between days 12 – 16 after the first day of menstruation. For females who reported cycle lengths longer than 28 days, the days of their HCR and LCR were adapted in response to their reported typical cycle length (counting back from the first day of menstruation [27]). That is, if a cycle was, say, 29 days, HCR would be calculated as beginning 29 – 16 days before the first day of menstruation.

Participants filled out a questionnaire to ensure they met inclusion criteria. If so, they were asked about the date of onset of their last menstrual period. If the participant indicated their onset had already occurred, forward counting from the day of onset allowed testing days to be scheduled for the days estimated most likely to be HCR and LCR. If the participant could not name the day of onset of their last period they were asked to notify the researcher when the next period began. From this information participants were randomly scheduled for their first testing session during either their estimated HCR or estimated LCR depending on which phase they were closest to upon successful completion of the questionnaire (see below). Seven females completed their first testing session during their estimated HCR (mean age = 25.23, S.D. = 7.43), and 5 females completed their first session during their estimated LCR (mean age = 25.2, S.D. = 6.91). Participants were instructed to indicate (via key press) whether they ‘liked’ the walk of each walker onscreen. Specifically, they were told, ‘regardless of your sexual orientation and regardless of whether you think the walker onscreen is a male or a female, do you like the way they walk?’ Key press was counterbalanced across participants. Participants were then scheduled to come in for the second testing session during their other conception risk phase and complete the same process. Before the start of the second session, participants were required to confirm where they were in their cycle to ensure accurate testing days were captured.

Participants in all experiments were instructed to press “M” or “Z”, and the alternatives were counterbalanced across participants in each experiment, to signal symmetrical/non-symmetrical walkers or female/male walkers. No feedback was given as to performance.

All experiments were designed as repeated measures studies, with each participant being tested on every condition. All analyses were designed for Repeated Measures ANOVAs with planed orthogonal contrasts. Data were analysed using the PSY programme (Professor Kevin Bird, UNSW). Target stimuli for these experiments were the -4 walker, the 0 walker, and the +4 walker, with others providing distractor stimuli to prevent repetition effects on the targets.

## Results

The challenge in testing gait preferences, as opposed to form or appearance preferences, is to eliminate, as far as possible, interactions between form and motion cues. With that in mind, the stimuli used here were variations of the so-called point-light walkers [28]. Preferences for symmetry in gaits have not previously been investigated using so-called point light display stimuli. As such, two preliminary experiments were run to confirm first that human female observers could detect differences in gait asymmetries in such displays and, second, that those differences did not affect their ability to discriminate the sex of individual walkers.

### Experiment 1

The aim of Experiment 1 was to test whether female observers could detect asymmetries in the gaits of point-light walkers used here. In particular, it was hypothesised that females would be able to discriminate low levels of FA in gait from high levels of FA.

**Figure 2:**
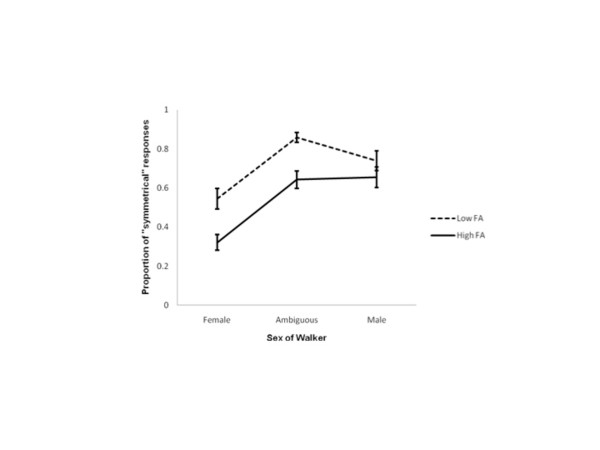
**Judgements of symmetry in point-light walkers with different levels of FA.** The proportion of “symmetrical” responses female observers reported for each walker. 1 indicates walkers were always reported to be symmetrical, 0 indicates walkers were never reported to be symmetrical, and 0.5 indicates that walkers were reported to be symmetrical on half the trials and asymmetrical on the remaining trials. Error bars represent ±1 standard error.

Data analyses were conducted on three point-light walkers only (extreme female, -4; gender ambiguous, 0; extreme male, +4), and for two levels of fluctuating asymmetry (low and high). These walkers were isolated to capture responses to unambiguously female gait, gender ambiguous gait, and unambiguously male gait respectively. For each of those walkers mean performances on each of the three stimuli of interest were computed for each individual participant, and then group means for each level of FA were calculated. Mean proportions of “symmetrical” judgments across these three walkers and two levels of FA are represented in Figure 2. As shown in Figure 2, female observers were able more often to discriminate the walkers with low FA as more ‘symmetrical’ than those with high FA: Gaits low in FA were reported to be symmetrical more often that gaits high in FA for the female walker (F_1, 25_ = 20.653, p < .000), ambiguous walker (F_1, 25_ = 22.607, p = .000) and male walker (F_1, 25_ = 8.884, p.006). Note, however, that none of the walkers tested were judged to be symmetrical 100% of the time, demonstrating that observers were able to discriminate even small levels of FA from gait.

Interestingly, for both low and high FA walkers there were differences between performances according to sex of the walker. For both FA levels, female walkers were judged to be symmetrical less often than either ambiguous or male walkers: Low FA walkers (F_1, 25_ = 7.053, *p* = .014), high FA walkers (F_1, 25_ = 18.010, *p* = .000). That is, female observers were more acute detectors of asymmetry in female walkers than in ambiguous or in male walkers (and pilot analyses showed the FA in female walkers equals that in the male walkers used here).

There could be a few possible reasons as to why this increased sensitivity to female gait was observed. Females may be more familiar with gait that is similar to their own, and thus, asymmetry in female gait may be more noticeable than asymmetry in male gait. Such an account would be consistent with the growing literature on mirror neurons [29]. Alternatively, females may be more critical of other females in general. Both alternatives represent interesting future research possibilities.

The aim of Experiment 1 was to test whether female observers could detect asymmetries in the gaits of point-light walkers. The data reported here show they can. Across all walkers, gaits low in asymmetry were reported to be ‘symmetrical’ more often than gaits high in asymmetry. No gaits were consistently reported to be symmetrical, and that is consistent with the physical characteristics of the stimuli: No walker had perfectly symmetrical gait. Thus, these results are consistent with previous studies showing observers could detect fluctuating asymmetries in faces. Additionally, female observers were better detectors of asymmetries in the female walkers than the ambiguous or male walkers.

### Experiment 2

Experiment 2 had the aim of determining whether females reliably could discriminate the sex of the walkers, independent of the level of asymmetry in their gait. Males and females are known to differ in physical morphology and dynamic motion, and others have shown that observers watching point-light display stimuli can discriminate the walker’s sex quickly and accurately from brief presentations [30], from different viewing angles [31], under altered lighting conditions [32], and from a range of actions [33]. Thus, the hypothesis tested in Experiment 2 was that females would be able to accurately discriminate the sex of the female and male walkers independently of gait asymmetries.

**Figure 3:**
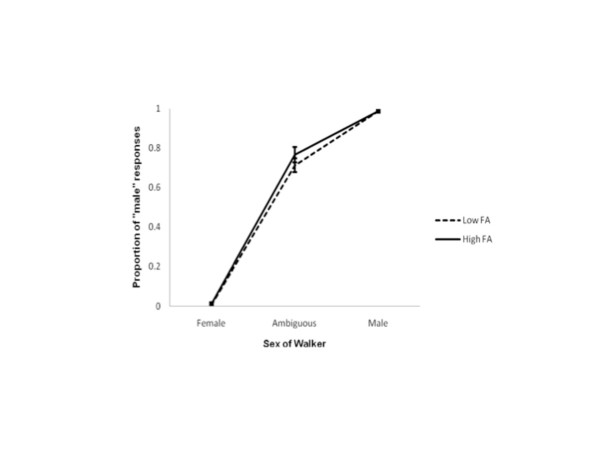
**Judgements of sex from point-light walkers with different levels of FA.** The proportion of “male” responses female observers reported for each walker. 1 indicates walkers were always reported to be male, 0 indicates walkers were never reported to be male, and 0.5 indicates that walkers were reported to be male on half the trials. Judgements for walkers with low and high levels of FA are shown separately. Error bars represent ± standard error.

Mean performances on each of the three stimuli of interest were again computed for each individual participant and then group means for each level of FA calculated. Mean proportions of “male” judgments across these three walkers and two levels of FA are represented in Figure 3. A one-way repeated measures ANOVA, with planned orthogonal contrasts revealed no effect of asymmetry on sex discriminations. There was no difference in observers’ abilities to discriminate sex in low and high FA female walkers (F_1, 25_ = 1.301, p = .265), ambiguous walkers (F_1, 25_ = 3.338, p = .080), or male walkers (F_1, 25_ = .000, p = 1.0). That is, female walkers were never reported to be male and male walkers were always reported to be male. Interestingly, for both levels of FA, ambiguous walkers were judged to be male more often than female, an example of the reliable male bias reported elsewhere [26,34]. Also, as predicted, there was a significant effect of sex for both levels of FA. The low FA male walker was reported to be “male” more often than either the low FA ambiguous and female walkers (F_1, 25_ = 11289.063, p = .000). Similarly, the high FA male walker was reported to be “male” more often than the high FA ambiguous and female walkers (F_1, 25_ = 8103.689, p = .000). Thus, it appears that FA does not affect acuity for sex discrimination for female observers.

The hypothesis that different levels of asymmetry in gait would not affect accurate sex discrimination was supported. Both the low and high FA female walkers were never reported to be ‘male’, and the low and high FA male walkers were always reported to be ‘male’. A “male-bias”, the tendency to perceive sexually ambiguous stimuli as male more often than female [34] was observed for both levels of asymmetry tested and there were no differences in the magnitude of that bias. Thus, it seems that sex perceptions are robust in the context of the different levels of FA used here. This is consistent with previous studies on sex discrimination where humans accurately judged the sex of walkers despite varying manipulations to gait [30-32].

### Experiment 3

The previous experiments revealed that female observers could detect a difference between gaits low in asymmetry and gaits high in asymmetry and confirmed that ability to successfully discriminate the sex of a walker is not disrupted by high asymmetries. Given that asymmetries were detectable, the sex of the walkers was identifiable, and that symmetry is preferred in structural traits when women are in their HCR phase [35], the aim of Experiment 3 was to investigate whether perceptions of gait change across the menstrual cycle. In particular, Experiment 3 tested whether there was a preference for symmetrical gait compared to less symmetrical gait. It was predicted that if symmetry in biological motion informs females’ mate selection preferences, female observers would prefer more symmetrical to less symmetrical gaits in males when judgements were made during HCR.

**Figure 4:**
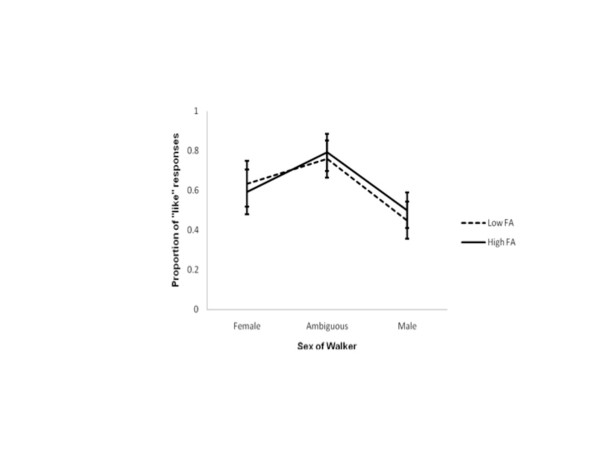
**“Liking” judgements during estimated LCR.** The proportion of “liking” judgements from female observers during their estimated LCR phase. 1 indicates the walker was reported as being liked on every presentation; 0 indicates the walker was never liked. Proportions for walkers with low and high levels of FA are shown separately. Error bars represent ± standard error.

**Figure 5:**
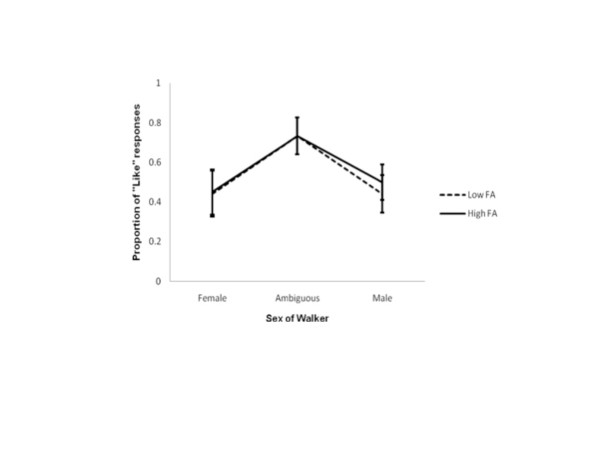
**“Liking” judgements during estimated HCR.** The proportion of “liking” judgements from female observers during their estimated HCR phase. 1 indicates the walker was reported as being liked on every presentation; 0 indicates the walker was never liked. Proportions for walkers with low and high levels of FA are shown separately. Error bars represent ± standard error.

Mean performances on each of the three stimuli of interest were computed for each individual participant and then group means for each level of FA calculated. Mean proportions of “liking” judgments across the three walkers and two levels of FA when female observers are in their low conception risk phase, and high conception risk phase are represented in Figures 4 and 5 respectively. A mean of 1 indicates walkers were always “liked”, a mean of 0 indicates walkers were never “liked”, and a mean of 0.5 indicates that walkers were “liked” on half the trials and “not liked” on the remaining trials. Error bars again represent standard errors.

“Liking” judgements at estimated LCR: As highlighted in Figure 4, when females were estimated to be in their low conception risk phase there was no difference in the rates of their liking judgements of low FA and high FA female walkers (F_1, 11_ = 2.570, p > 0.05), of ambiguous walkers (F_1, 11_ = 0.449, p > 0.05), or of male walkers (F_1, 11_ = 0.347, p > 0.05). In other words, these data suggest there are no differences in female preferences for low or high FA gaits in walkers of any sex at estimated LCR.

“Liking” judgements at estimated HCR: Figure 5 shows the proportion of “liking” judgements from female observers estimated to be in their high conception risk phase. Similar to the trends observed at estimated LCR, at estimated HCR there was no difference in female observers’ liking judgements of low FA and high FA female walkers (F_1, 11_ = 0.048, p > 0.05), of ambiguous walkers (F_1, 11_ = 0.000, p > 0.05), or of male walkers (F_1, 11_ = 0.789, p > 0.05). In other words, these data suggest there are no differences in female preferences for low or high FA gaits in walkers of any sex at estimated HCR.

**Figure 6:**
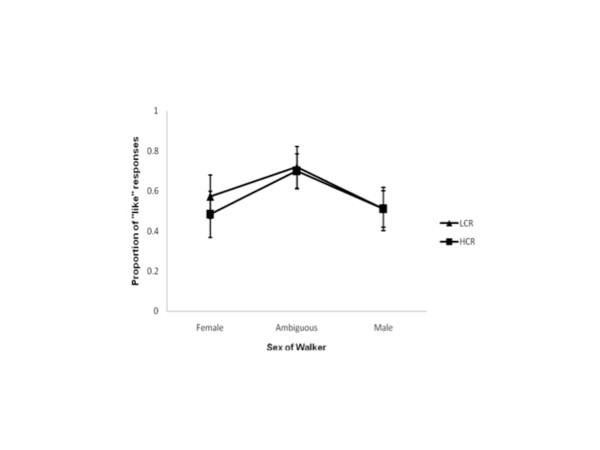
**“Liking” judgements independent of FA.** The proportion of “liking” judgements from female observers at both their estimated LCR and HCR phases independent of the level of FA present in the walkers. 1 indicates the walker was reported as being liked on every presentation; 0 indicates the walker was never liked. Proportions at estimated LCR are shown separately from proportions at HCR. The difference for the female walkers is significant. Error bars represent ± standard error.

Given that there was no effect of asymmetry on observers’ liking judgements data were collapsed across levels of FA, and a post-hoc one-way repeated measures ANOVA was run to investigate whether there was any differences in the way females evaluated gait across the menstrual cycle independent of asymmetry. Figure 6 shows the mean proportions of “liking” judgements at each conception risk phase tested. As shown there, female observers liked the female walker more at estimated LCR than estimated HCR, (F_1, 11_ = 8.520, p ≤ 0.017; Bonferroni adjusted at α = 0.05, k = 3). Most interestingly, there was no difference in liking judgements across conception risk phases for the ambiguous walker (F_1, 11_ = 0.579, p > 0.017) or male walker (F_1, 11_ = 0.008, p > 0.017). That is, female observers did not differ in their preferences for male gaits as their risk of conception varied. They did, however, show decreased preferences for the gaits of other females as they moved from low conception risk to high.

Thus, in summary, these data do not support the hypothesis that females at high conception risk would prefer male gait that was more symmetrical to gait that was less symmetrical. Instead, these data suggest changes in hormonal state relating to conception risk affect female perceptions of other female’s biological motions rather than their perceptions of male biological motions. Of course, these findings need replicating. This was an unexpected result and should directly be investigated in future work.

## Discussion

The general aim of this research was to investigate whether human females experienced changes in their preferences for walking gaits across the menstrual cycle. In particular, it was predicted that female observers would show preferences for male gaits with low levels of FA during the estimated HCR phase of their menstrual cycle compared to judgements made during their estimated LCR phase. To test that hypothesis point-light walkers with low and high levels of FA were constructed and female observers were asked first to discriminate low from high levels of FA, and they could do that reliably (Experiment 1). Indeed, females were more acute observers of asymmetries in the gaits of other females than they were of asymmetries in male gaits. Those same observers were asked also to discriminate female from male walkers with both low and high levels of FA and they could do that reliably also (Experiment 2). Surprisingly, and contrary to predictions, a sub-set of the observers showed no changes in preferences for low and high FA across their menstrual cycles (Experiment 3). That is, conception risk did not predict female preferences for male gaits with low FA: Low FA walkers were never preferred to high FA walkers in either conception risk phase. This finding contrasts the prediction that females would prefer male walks that are more symmetrical at HCR. Interestingly, however, it was found that independent of FA, female observers liked the gaits of other female walkers less during the estimated HCR phase of their menstrual cycle compared to judgements made during their estimated LCR phase. That same variation was not evident in judgements of non-female walkers.

While not predicted, that result is not without precedent. It has been shown previously that females at peak fertility prefer less the faces of other females. That is, women show decreased preferences for feminine faces when progesterone levels are low and oestrogen levels are high [3]. Jones and his colleagues suggested that variation in “preferences for feminine female faces may be a functionless (but low cost) by-product of attraction to cues to commitment or immunity in males” (p. 289). While the data reported here do not provide a strong counter example, that females show, across their menstrual cycle, variations in their preferences not just for feminine faces but also for feminine gait suggests the effect has a purpose. Gangestad and Cousins have argued that such behaviours might reflect increased competition between females for strong mates at times when at least one of the females is likely to fall pregnant if intercourse occurs [36]. If that is so, sensitivity to cues that predict which other females might represent the strongest competition during periods of peak fertility might give an individual female a competitive advantage. Of course, that hypothesis requires testing.

What is clear from these data is that the levels of FA in the gaits used in these experiments do not interact with variations in fertility: Even though female observers could discriminate between female gaits showing low and high levels of FA, the decrement in “liking” for the gaits of other females was independent of level of asymmetry being displayed. Similarly, there were no variations observed here in preferences for male gaits with low and high levels of FA across the menstrual cycle. Those observations suggests that while female observers are sensitive to gait asymmetries, especially in other females, they do not use that particular cue in the same way they use structural cues to health or genetic robustness. As noted above, there is a substantial literature indicating females do vary their preferences for faces and other features correlated with health-related traits as fertility varies.

### Limitations

It is tempting to speculate that gait asymmetries are not among those. Of course, that argument needs to be tempered with the observation that the walkers used here are not individuals but average walker. Similarly, the asymmetries added to these stimuli are not natural in that the fluctuating asymmetries in our stimuli are based on changes between averages. That means that before stronger conclusions can be drawn it may be necessary to test the effects reported here with real asymmetries in real walkers. Doing so may recruit discrimination mechanisms not sensitive to the manipulations used here.

That there were no interaction between FA and fertility seen here could be because symmetry in motion may not be as reliable a cue to the (genetic) characteristics on an individual as symmetry in static, structural traits like faces, bodies, and scents. Symmetry in structural traits does not usually vary, and under normal circumstances, cannot be ‘faked’ [11]. Contrastingly, symmetry in gait can and does vary either through voluntary or involuntary mechanisms. For example, gait change according to mood and emotional state and observers reliably can tell the difference between the gaits of those who are happy or confident and those that are vulnerable [37]. Similarly, carrying objects can affect gait and “fit” individuals potentially can carry heavier objects than those that are less fit. In doing so those that are stronger might be more likely to exhibit an asymmetry while doing so. Finally, even the terrain over which an individual is walking can affect the symmetry of gait, suggesting perhaps that judgements of gait “balance”, rather than symmetry, might be a more reliable cue to the genetic health of an individual.

## Conclusions

This study contributes some innovations to research on human mate preferences and the underlying mechanisms that drive such processes. This study indicates that female observers may not value gait asymmetry as a mate selection cue in the same way that they value symmetry in faces and bodies. In summary, these data show female observers are capable of discriminating the sex of individuals on the basis if their gait and that they can do so across different levels of fluctuating asymmetry. These data show too that the magnitudes of those asymmetries do not affect female observers’ preferences for individuals as fertility varies across the menstrual cycle. Thus, asymmetries in the motion cues used here are not used in the same way as static cues for judging the apparent healthiness of individuals, female or male. Interestingly, female observers did changes in their preferences for other females, on the basis of their gaits, across their menstrual cycles. Females showed decreased preferences for other females at times when it was estimated that the observers were at peak fertility. While the neural mechanisms mediating those behaviours may be both unconscious (and currently unknown) they may equip individual females to act in ways that would increase opportunities for mating with strong males at times of peak fertility.

## Competing interests

Both authors declare that they have no competing interests.

## Authors’ contributions

NH carried out the data collection and preliminary treatment of all data. vdZ designed the study and with NH performed the statistical analysis. NH and vdZ conceived of the study, and both drafted the manuscript. vdZ supervised the project. All authors read and approved the final manuscript.
